# Altered Neural and Behavioral Response to Sexually Implicit Stimuli During a Pictorial-Modified Stroop Task in Pedophilic Disorder

**DOI:** 10.1016/j.bpsgos.2022.02.004

**Published:** 2022-02-24

**Authors:** Christian Mannfolk, Benny Liberg, Christoph Abé, Christoffer Rahm

**Affiliations:** a Centre for Psychiatry Research, Department of Clinical Neuroscience, Karolinska Institutet, and Stockholm Health Care Services, Region Stockholm, Sweden; b Department of Clinical Neuroscience, Karolinska Institutet, Stockholm, Sweden

**Keywords:** Attention, Default mode network, Executive function, Functional magnetic resonance imaging, Pedophilia, Stroop task

## Abstract

**Background:**

Pedophilic disorder (PD) entails sexual attraction to prepubertal children. A risk factor for committing child sexual abuse in PD is impaired cognitive control. However, the underlying neurocognitive mechanisms remain unclear.

**Methods:**

We performed a case-control study including 51 self-identified and help-seeking males with PD and 55 matched healthy control subjects. Functional magnetic resonance imaging and a pictorial-modified Stroop task involving computer-generated sexually implicit images were used to measure response time and brain activation. Increases in response time during the pictorial-modified Stroop task are presumably due to image-induced interference in executive functions required for task performance.

**Results:**

In PD, during the presentation of images of children compared with adults, we found increased response time (*p* = .005; 848 ± 92 ms vs. 826 ± 88 ms), and compared with healthy control subjects, we found increased activation in the occipital, temporal (bilateral hippocampus), parietal, frontal, cingulate, and left insular cortices; caudate (bilaterally); thalamus (mediodorsal); and cerebellum.

**Conclusions:**

Presentation of child images was associated with response interference in PD and increased engagement of brain regions involved in the processing of sexual stimuli, visual perception, self-referential thought, and executive function. We conclude that processing of child images is associated with functional and behavioral alterations in PD.

Pedophilic disorder (PD) refers to a persistent sexual attraction to prepubertal children that leads to associated actions and/or marked mental distress (1). Individuals with PD have an increased risk of committing child sexual abuse (CSA) (2). However, only 50% of convictions for CSA are estimated to be committed by perpetrators who fulfill criteria for PD (2). To prevent CSA, it is essential to determine the endogenous cognitive processes that underlie the development of altered behavioral control in PD. PD is associated with lower occupational status, higher prevalence of left-handedness, and lower IQ (3,4). Cognitive deficits include verbal fluency, emotion perception, empathy, executive function, working memory, and attention (5,6). Poor executive function contributed to an elevated CSA risk in a help-seeking clinical PD sample (7,8) and has been correlated to convictions for contact CSA offenses in a forensic PD sample (9,10). The Stroop task is a cognitive paradigm used to study executive functions such as cognitive control, response inhibition, and the ability to inhibit cognitive interference (11,12). These functions often rely on the ability to solve cognitive conflicts, manage distractors, and inhibit attention bias, e.g., in the presence of a distracting stimulus (13).

The pictorial-modified Stroop task (P-MST) is a modified version of the original Stroop task (14). The P-MST was first published by Ó Ciardha and Gormley using a sample including CSA offenders (14), leaning on previous work in the field (15). In the P-MST, sexually salient distractors compete for attention and interfere with executive attention during goal-directed behavior. Sexual interference results in a sexual content–induced delay (SCID) in behavioral response time (RT), which presumably relates to attention bias and thus the ability to maintain cognitive control (16). Executive function, which includes cognitive control, is dependent on frontal lobe function (17).

Neural models of sexual content–related information processing in PD broadly suggest the involvement of large-scale neuronal networks related to sexual desire and executive functions. These networks are composed of the frontal lobe (lateral prefrontal cortex, orbitofrontal cortex, medial frontal cortex), subcortical limbic regions (hypothalamus, periaqueductal gray), paralimbic regions (insula and anterior cingulate cortex), striatum, thalamus, and cerebellum (18, 19, 20). Functional brain imaging studies of PD have predominantly focused on processing and perception of preferential sexual stimuli in convicted offenders (21, 22, 23, 24, 25, 26, 27, 28, 29, 30, 31).

One previous study has investigated cognitive control with sexual distractors (32), and two without sexual distractors (9,33), in PD. Although these studies had small sample sizes, they suggest that during task performance with increased executive attention demands, there is increased activation of paralimbic regions (insula; cingulate cortex, anterior and posterior division; precuneus), prefrontal cortex, and parietal cortex (angular gyrus), independently of sexual distractors. However, the P-MST task, including sexual salient images, has never been applied in a nonforensic sample of self-identified and help-seeking individuals with PD at risk of committing CSA, and the neural correlates of behavioral control in PD remain unknown (8). This, and the characterization of the at-risk state of committing CSA, could help identify predictors for CSA and treatment targets aimed at preventing CSA.

The primary goal of this study was to determine whether there are alterations in the neural activation of brain regions involved in behavioral control during the P-MST in individuals with PD at risk of committing CSA. We hypothesized that attention bias to sexually implicit stimuli leads to disengagement of executive attention during goal-directed behavior in individuals with PD at risk of CSA. We made two main predictions. The first prediction was that preferential and sexually salient stimuli induce SCID and therefore RT delay. More specifically, SCID for child images should be exclusive to participants with PD, whereas SCID for adult images should be strong among healthy control (HC) subjects and somewhat weaker among participants with PD. SCID is also expected to depend on the congruence of stimuli sex with the participant’s sexual orientation. For example, a male heterosexual HC participant should have the strongest SCID when viewing images presenting adult females. The second prediction was that the level of neural activation during task performance will differ between participants with PD and control subjects, primarily in frontal brain regions involved in executive function. A counting Stroop was included in the experiment to provide a cognitive control measure unrelated to sexual stimuli processing. Within a case-control study design, we used functional magnetic resonance imaging (fMRI) and behavioral measures (RT) from the P-MST to investigate our hypotheses.

## Methods and Materials

### Participants

**Figure 1:**
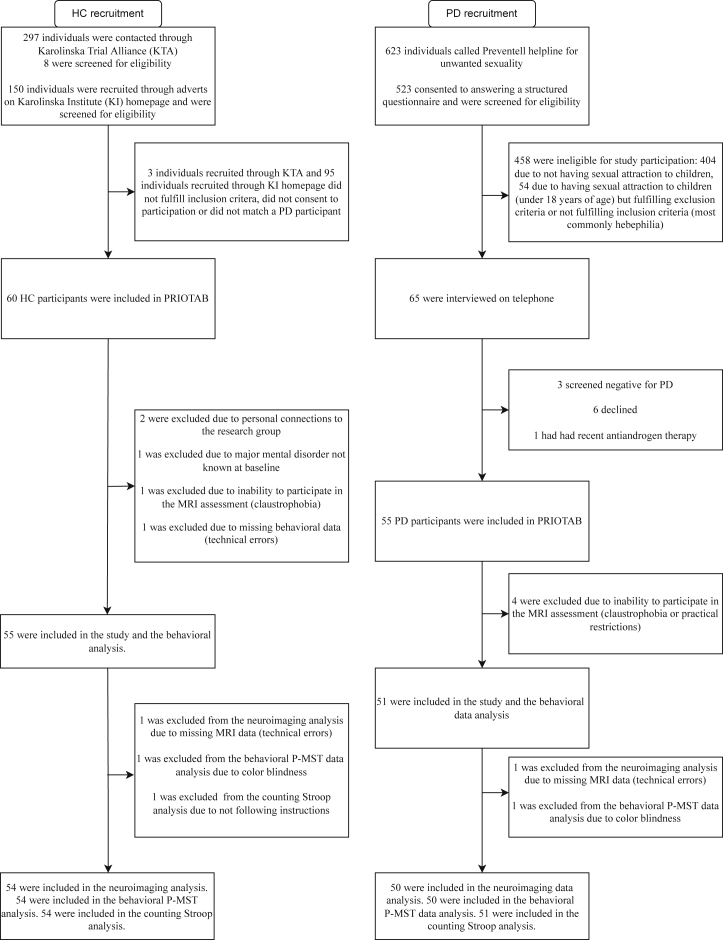
Recruitment flowchart. A flowchart visualizing the recruitment procedure for the healthy control (HC) participants and participants with pedophilic disorder (PD) in the study. KI, Karolinska Institute; KTA, Karolinska Trial Alliance; MRI, magnetic resonance imaging; P-MST, pictorial-modified Stroop task; PRIOTAB, Pedophilia at Risk - Investigations of Treatment and Biomarkers.

Between March 2016 and April 2019, 51 men with PD were recruited from a Swedish national helpline for unwanted sexuality along with 55 age- and sex-matched HC participants recruited via public advertisements. Inclusion criteria were age between 18 and 66 years and male. Exclusion criteria were MRI contraindications and severe psychiatric comorbidity, such as schizophrenia. An additional inclusion criterion for the patient group was a PD diagnosis verified in a screening interview by a specialist psychiatrist, and exclusion criterion was any contraindication to degarelix, because patient participation also entailed inclusion in a degarelix trial (for more information regarding the trial, see elsewhere), with treatment start occurring after data gathering finished for this study (34). A flow diagram visualizing the recruitment and inclusion process is shown in Figure 1. Subjects underwent psychiatric screening for clinical characterization, including clinical interview, neuropsychological test battery, and several questionnaires; for the sample characteristics, see Table 1. Effect sizes for Table 1 were estimated with Cohen’s *d*, calculated as standardized mean differences for parametric data via biserial rank correlations for nonparametric data, or from frequency distributions (in a 2 × 2 frequency table) for categorical data. For more details about the recruitment procedure and participant characterization, see the Supplement, Sections A and B. A total of 50 participants with PD and 54 HC participants provided neuroimaging data of sufficient quality for the fMRI analysis (for details, see Figure 1, Table 2, or Section B of the Supplement). All participants provided written informed consent to participate in the study. The National Swedish Ethics Review Appeals Board approved the study protocol (Ö 26–2014).

**Table 1:** Study Participant Characteristics

Characteristics	PD, *n* = 51	HC, *n* = 55	Cohen’s *d*; *p* Value
Age, Years, Range, Mean (SD)	18–66, 35.8 (11.4)	18–64, 36.4 (11.8)	−0.05; .792
Education, Highest Level, *n* (%)			
Primary school ≤9 years	5 (10%)	2 (4%)	−0.30; .303
Secondary school 1–3 years	24 (47%)	23 (42%)	
Postsecondary education	22 (43%)	30 (55%)	
Employed, *n* (%)	31 (61%)	48 (87%)	−0.82; <.001^a^
Parent, *n* (%)	19 (37%)	25 (46%)	−0.19; .392
Currently Lives as Single, *n* (%)	34 (67%)	20 (36%)	0.69; .002^a^
Ever Lived With Partner for More Than 2 Years, *n* (%)	25 (49%)	40 (73%)	−0.56; .012^a^
RAADS-14, Range, Median (IQR)^b^	0–39, 18 (19)^c^	0–27, 4 (4)	1.03; <.001^a^
RAADS-14 screening cutoff, total score >13, *n* (%)	31 (62%)^c^	5 (9%)	1.34; <.001^a^
Full-Scale IQ, WAIS-IV, Median (IQR)^b^	101 (23)^d^	115 (16)^e^	−0.82; <.001^a^
WAIS-IV processing speed (WAIS-IV speed), median (IQR)^b^	101 (19)^d^	106 (19)^e^	−0.48; .016^a^
ASRS Positive Screening, >3, *n* (%)	18 (36%)^c^	9 (16%)	0.58; .021^a^
HBI, Range, Median (IQR)^b^	19–93, 56 (22)^c^	19–54, 26 (10)	2.13; <.001^a^
HBI screening indicative of hypersexuality, ≥53, *n* (%)	31 (62%)^c^	1 (2%)	2.47; <.001^a^
SDI, Range, Median (IQR)^b^	26–100, 70.5 (22)^c^	38–83, 64 (14)^f^	0.21; .283
SDI screening indicative of hyposexuality, <46, *n* (%)	7 (14%)^c^	2 (4%)^f^	0.79; .066
AUDIT, Range, Median (IQR)^b^	0–27, 2 (5)^c^	0–15, 5 (3)	0.30; .003^a^
AUDIT total score >7, *n* (%)	6 (12%)^c^	9 (16%)	−0.20; .523
DUDIT, Range, Median (IQR)^b^	0–12, 0 (2)^c^	0–8, 0 (0)	−0.17; .395
DUDIT total score >5, *n* (%)^g^	4 (8%)^c^	2 (4%)	0.46; .421
Edinburgh Handedness Inventory (Swedish Version), Right Handedness, *n* (%)	41 (80%)	43 (78%)	−0.07; .779
Antidepressive Medication, *n* (%)	14 (28%)	3 (6%)	1.04; .002^a^
Other Psychiatric Medication, *n* (%)	12 (24%)	3 (6%)	0.92; .007^a^
Heterosexual Preference for Adults, *n* (%)^h^	28 (72%)	43 (83%)	−0.35; .214
Pedophilic Sexual Attraction			
Age of discovery of pedophilic sexual attraction, range, mean (SD)^i^	6–39, 18.7 (7.4)	NA	–
Attraction to boys, *n* (%)	7 (14%)	NA	–
Attraction to girls, *n* (%)	40 (78%)	NA	–
Attraction to boys and girls, *n* (%)	4 (8%)	NA	–
Attraction exclusively to prepubescent children, *n* (%)	12 (24%)	NA	–
Self-reported Convictions, *n* (%)			
Any sexual offense	12 (24%)	1 (2%)	1.52; <.001^a^
Any contact sexual offense^g^	5 (10%)	NA	0.99; .023^a^
Any noncontact sexual offense^g^	8 (16%)	NA	1.28; .002^a^
Any nonsexual offense	8 (16%)	11 (20%)	−0.02; .563

Demographic, criminal, and sexual characteristics for PD and HC participants. For reasons of completeness, we exchanged a frequency of 0 to 1 in effect size calculations to obtain Cohen’s *d* estimates.

ASRS, ADHD Self Report Scale; AUDIT, Alcohol Use Disorder Identification Test; DUDIT, Drug Use Disorder Identification Test; HBI, Hypersexual Behaviour Inventory; HC, healthy control; IQR, interquartile range; NA, not applicable; PD, pedophilic disorder; RAADS-14, Ritvo Autism and Asperger Diagnostic Scale-14; SDI, Sexual Desire Inventory; WAIS-IV, Wechsler Adult Intelligence Scale-IV.

a Statistically significant at *p* < .05 (two-tailed).

b Cohen’s *d* was calculated from the Mann-Whitney *U* test, because these metrics were nonparametric, except for WAIS-IV speed, where Cohen’s *d* was calculated in the same way as for full-scale IQ (WAIS-IV).

c Missing data from 1 participant with PD.

d Four participants with PD did not complete WAIS during their first visit; instead, the value from the second or third visit was used.

e Missing data from 1 HC participant.

f Missing data from 2 HC participants.

g Expected counts were <5 in one or more cells, and *p* values were calculated with Fisher’s exact test.

h Missing data from 12 participants with PD exclusively attracted to children, in addition to 3 HC participants.

i Excluded 3 participants reporting that they “had always known” about their pedophilic sexual attraction and who did not provide a specific age.

**Table 2:** Participant Characteristics.

Characteristics	PD			HC*, n* = 54
	Total, *n* = 50	Exclusive^a^*, n* = 11	Nonexclusive^a^*, n* = 39	
Age, Years, Mean (SD)	35.8 (11.6)	40.9 (14.9)	34.3 (10.2)	36.7 (11.7)
Bisexual^b^, *n*	9	1	8	5
Homosexual^b^, *n*	6	2	4	4
RAADS-14 Score >13, *n* (%)	31 (62%)	8 (73%)	22 (58%)	5 (9%)
WAIS-IV Processing Speed (WAIS-IV Speed), Mean (SD)	100 (13.3)	105 (17.4)	99 (11.8)	106 (12.3)^c^

Characteristics of PD and HC participants in the main fMRI analysis.

fMRI, functional magnetic resonance imaging; HC, healthy control; PD, pedophilic disorder; RAADS-14, Ritvo Autism and Asperger Diagnostic Scale-14; WAIS-IV, Wechsler Adult Intelligence Scale-IV.

a Exclusivity with regard to pedophilic interest, i.e., exclusively attracted to children or not.

b The sexuality presented here is simply based on the self-reported sexually preferred sex, regardless of if that preference was pedophilic or not. Therefore, a participant who prefers children of the female sex and adults of the male sex will be classified as bisexual. Grouping or exclusion according to sex preferences was necessary for one of the secondary analyses presented in the Supplement. We chose to use the nomenclature of bi-, homo-, and heterosexual for clarity and readability.

c One HC participant did not participate in WAIS-IV.

### Experimental Paradigm

**Figure 2:**
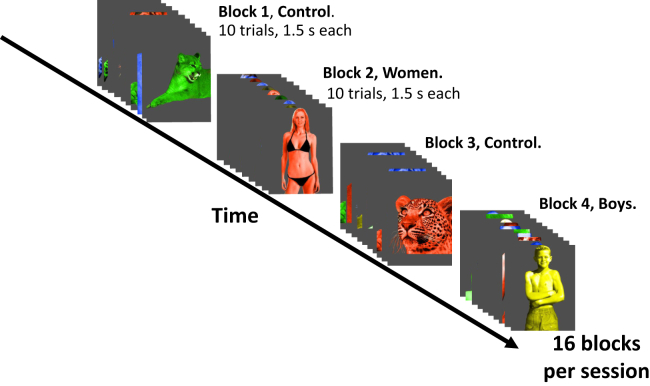
Illustration of the pictorial-modified Stroop task block design.

The P-MST test paradigm was generously provided by Ó Ciardha and Gormley (14). In the P-MST, there were five different stimuli categories presented to the participants: images depicting cats (control stimuli), men (Tanner stage 5, i.e., adults), women (Tanner stage 5), boys (Tanner stage 1, i.e., prepubertal), and girls (Tanner stage 1). Subjects were instructed to respond as quickly and accurately as possible to what color tint the picture had, by pressing one of four corresponding buttons on a response device. The pictures were computer-generated (adult and child) images of nonreal individuals that are part of the Not Real People stimulus set (35,36), modified for use in the P-MST, alongside (cat and adult) images created for the original P-MST study (14,37). The human pictures are shown in a frontal view while only wearing swimwear. The P-MST experiment involved a block design, where each block contained only boys, girls, men, women, or control stimuli and had a duration of 15 seconds (10 trials presented for 1.5 s each). Every second block was a control block. There were two sessions conducted in succession, with each session containing 16 blocks, including two blocks of each human stimuli category (boys, girls, men, women) and eight control blocks. At the beginning and end of each session, there was a 30-second period with a fixation spot. The block design is visualized in Figure 2. The stimulus presentation order was pseudorandomized and differed between the sessions. Only human stimuli were included in the analysis, separated by image type (adult = men + women vs. child = boys + girls). The control pictures (cats) were omitted from the analysis (but were used in a post hoc analysis presented in the Supplement) because for our research question, (wild) animal image content was unsuitable for comparison with human images (38, 39, 40). For the counting Stroop, participants were instructed to answer with the number of copies of a presented word. Words were either neutral or incongruent and were presented in a block design. Incongruent words are number words not congruent with the number of copies presented, for example, two copies of the word “three”. For more details regarding the P-MST and the counting Stroop paradigm, see the Supplement, Section C.

### Brain Imaging

A Siemens Prisma 3T scanner was used to acquire blood oxygen level–dependent sensitive T2∗-weighted echo planar images. Each imaging volume comprised 42 axial slices with an isotropic resolution of 3 × 3 mm (slice gap, 3 mm; echo time, 34 ms; field of view, 240 × 240 mm; flip angle, 90°) covering the whole brain. Volumes were acquired with a repetition time of 2.5 seconds. The first six (dummy) volumes of each run were discarded to allow for T1 equilibration effects. A total of 120 volumes were acquired during each of the two runs. Imaging data were analyzed using the FSL 6.0.1 software suite with further brain imaging details presented in the Supplement, Section D (41).

In brief, the subject-specific contrast (contrast of parameter estimates) images of the effect of interest from both runs were entered into a second-level fixed-effects analysis to create centrally ordered phase encoding images of mean activation across runs. For each individual, the main contrast maps investigated were created by subtracting brain activity maps under the adult image type from maps under the child image type (child > adult). The resulting centrally ordered phase encoding images of mean activation were fed into a third-level analysis of within-group mean activation and between-group differences. Mean RT difference (ΔRT = RT_Adult_ − RT_Child_) was added separately in an additional regression model to determine its effects (and group × ΔRT interaction) on neural activation. *Z* (Gaussianized T/F) statistic images were thresholded using clusters determined by *Z* ≥ 3.1 and a cluster significance threshold (corrected using Gaussian random field theory) of *p* ≤ .05. Sensitivity analyses conducted on the fMRI results are presented in Section E of the Supplement.

### Analysis of Behavioral Data

In the P-MST, we hypothesized that patients with PD would show SCID when exposed to child stimuli and HC subjects during adult stimuli. Therefore, we expected a group × image type interaction, and in the main behavioral analysis we tested for the main effects of image type (adult vs. child; within-subject factor), group (PD vs. HC), and group × image type interaction (main variable of interest) on RT using a 2 × 2 mixed factorial analysis of variance. Data were analyzed with IBM SPSS 25.

Significant group × image type interactions were followed up with group comparisons in RT differences (ΔRT = RT_Adult_ − RT_Child_) between the child and adult image types (corresponding to the main fMRI contrast) using univariate analysis of variance. The PD group was expected to show negative ΔRT (SCID under the child image type), whereas HC subjects were expected to show positive ΔRT (SCID under the adult image type). Complementary paired *t* tests (RT_Adult_ vs. RT_Child_) were performed to test whether the observed changes in RT were significant within each group. Effect sizes of mean differences were expressed in Cohen’s *d*. All significant results were tested for effects of participants’ age. A secondary analysis investigated RT changes relative to RT_Adult_ performance (percent-RT = ΔRT/RT_Adult_). Secondary analysis tested how exclusiveness (regarding the pedophilic interest of patients with PD) influenced the findings by comparing behavioral outcomes between exclusive (stated attraction exclusively to children and not adults) and nonexclusive PD (stated attraction to both children and adults). Further secondary analysis was carried out to test the influence of congruence between participants’ sexual orientation and stimuli sex on RT (P-MST orientation congruence analysis). Results indicating a significant group difference in the overall RT in the P-MST led to a secondary post hoc follow-up analysis to determine the influence of the child RT on the significant group difference. The method for these secondary analyses is presented in Section E of the Supplement.

In the counting Stroop, we aimed to determine differences in overall performance between the groups. For further information about data preprocessing and the counting Stroop analysis, see the Supplement, Section E.

## Results

### Behavioral Results

**Figure 3:**
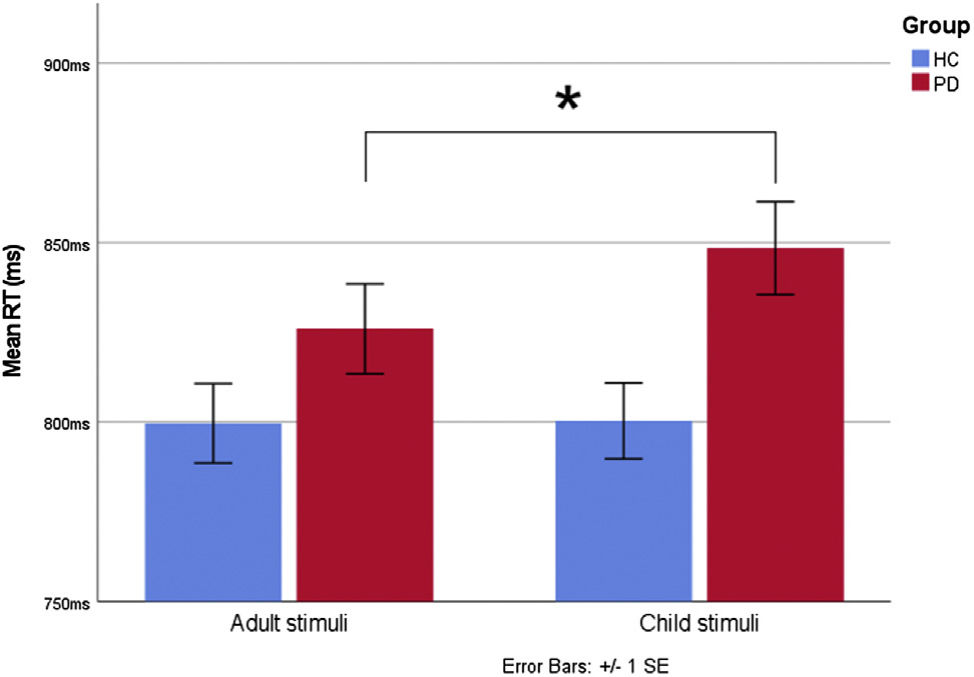
Mean response time (RT) in the pictorial-modified Stroop task for participants in the pedophilic disorder (PD) and healthy control (HC) groups for the adult and child image types. ∗ indicates a statistically significant difference (*p* < .05).

We found significant effects on RT from image type (RT_Adult_ = 812.80 ± 84.87 ms; RT_Child_ = 824.36 ± 84.81 ms; *F*_1,102_ = 6.399, *p* = .013, η_p_^2^ = 0.059), group (RT_HC_ = 799.97 ± 81.52 ms; RT_PD_ = 837.20 ± 81.52 ms; *F*_1,102_ = 5.415, *p* = .022, η_p_^2^ = 0.050), and the group × image type interaction (*F*_1,102_ = 5.689, *p* = .019, η_p_^2^ = 0.053). Groups differed significantly in ΔRT (mean ΔRT_PD_ = −22.44 ms, SD = 54.61 ms; mean ΔRT_HC_ = −0.66 ms, SD = 37.56 ms; *F*_1,102_ = 5.689, *p* = .019, η_p_^2^ = 0.053). Similar results were obtained for the relative percent change in RT (see Section F of the Supplement). Paired *t* tests revealed that the differences in RT between image types were significant within the PD group (mean RT_Adult_PD_ = 825.97 ms, SD = 88.46 ms; mean RT_Child_PD_ = 848.42 ms, SD = 91.58 ms; Cohen’s *d* = 0.41, *p* = .005, *t*_49_ = −2.906) but not in the HC group (mean RT_Adult_HC_ = 799.63 ms, SD 81.28 ms; mean RT_Child_HC_ = 800.30 ms, SD = 77.89 ms; Cohen’s *d* = 0.02, *p* = .898, *t*_53_ = −0.129). RT means for each group and image type for this analysis are plotted in Figure 3.

In secondary analyses (see Section F of the Supplement), the exclusive PD subgroup showed the most pronounced child SCID effect. The interaction between sexual orientation and stimuli sex had no effect on RT (and SCID). There was no significant difference in overall mean RT between the groups in the counting Stroop. The post hoc analysis indicated that the significant overall group RT difference in the P-MST did not survive when adjusting for the influence of the PD child SCID.

### Whole-Brain Neural Activation at the Cluster Level

**Figure 4:**
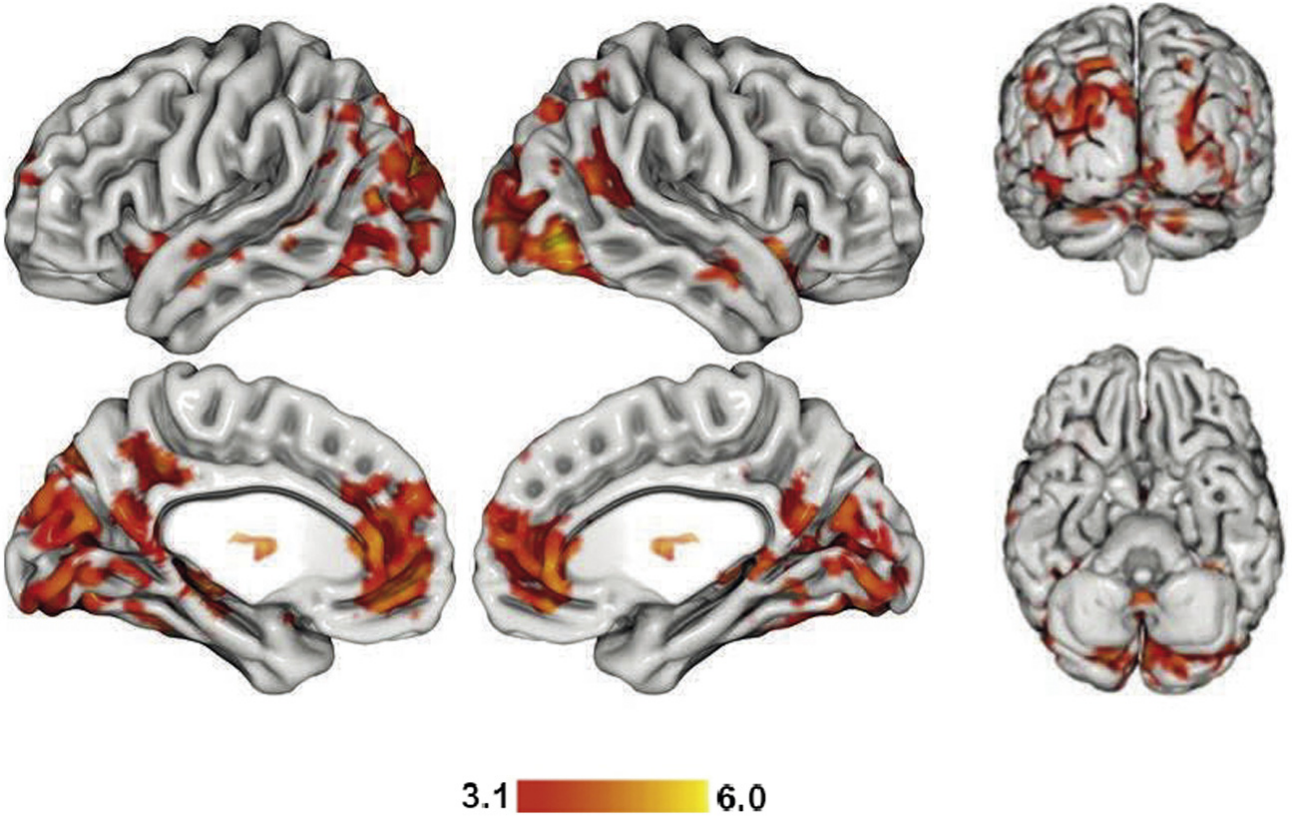
Functional magnetic resonance imaging group differences. Group differences in the pictorial-modified Stroop task–related mean activity (child > adult) between participants with pedophilic disorder and healthy control participants. Warm colors indicate regions where participants with pedophilic disorder showed significantly greater activation than healthy control subjects (pedophilic disorder > healthy control). *Z* values are indicated by the color bar.

Greater neural activation in PD patients compared with HC subjects in the main contrast comprised nine clusters (Figure 4 and Table S1). Broadly, cluster 9 (*Z*-max = 6.04) included the visual cortex and hippocampus subiculum; cluster 8 (*Z*-max = 4.99) included the anterior cingulate cortex and thalamus; cluster 7 (*Z*-max = 4.77) was confined to the angular gyrus; cluster 6 (*Z-*max = 5.8) included the insular cortex, lateral temporal cortex, and temporal pole; cluster 5 (*Z-*max = 4.58) included the orbitofrontal cortex, insula, and temporal pole; cluster 4 (*Z-*max = 4.27) included the premotor cortex and medial frontal cortex; cluster 3 (*Z-*max = 4.13) included the lateral temporal cortex and temporal pole; cluster 2 (*Z-*max = 4.96) included the primary somatosensory cortex; and cluster 1 (*Z-*max = 4.92) included the orbitofrontal cortex and frontal pole. Comparison of significant between-group activation of each cluster survived correction for each clinical characterization variable of interest (see Supplement, Section E for procedure and Section G for results).

### Whole-Brain Neural Activation at the Voxel Level

A detailed evaluation of within-cluster activations (local maxima presented in Table S2) revealed that the PD group showed greater neural activation during the main contrast (child > adult) as compared with the HC group in the occipital cortex (lateral occipital cortex and occipital fusiform), temporal cortex (bilateral hippocampus; left middle and superior temporal gyrus, anterior and posterior division; and left temporal pole), parietal cortex (right angular gyrus, left postcentral gyrus), frontal cortex (medial and frontal poles, bilaterally; right middle frontal gyrus, right precentral gyrus; and right inferior frontal gyrus, pars opercularis), cingulate cortex (anterior and posterior division), insular cortex (left), caudate (bilaterally), thalamus (mediodorsal), and cerebellum (posterior lobe and vermis). HC subjects did not show increased activation during the main contrast as compared with participants with PD. We found no statistically significant effect of ΔRT (or group × ΔRT) on neural activation. Mean group activation for the child > adult contrast within the PD and HC groups are presented in Figures S4 and S5 in the Supplement, respectively.

## Discussion

In this study, we have demonstrated altered neural (blood oxygen level–dependent) activation and behavioral response to child > adult stimuli in the P-MST for participants with PD compared with HC subjects. On the behavioral level, we found that patients with PD displayed a SCID effect during presentation of child images. Adult images did not cause SCID for either group, which was unexpected for HC subjects. In addition, SCID was not influenced by sexual orientation and stimuli sex. In the child > adult contrast, compared with HC subjects, patients with PD showed increased fMRI engagement in the occipital, temporal (bilateral hippocampus), parietal, frontal, cingulate, and left insular cortices; caudate (bilaterally); thalamus (mediodorsal); and cerebellum (posterior lobe and vermis).

### Implications From the Behavioral Results

PD displayed SCID during presentation of child stimuli indicative of attention bias induced by child stimulus content. Our secondary analysis suggested that child SCID (negative ΔRT) in the PD group was driven by the subgroup of those with exclusive pedophilic interest (Section F of the Supplement). However, these results should be interpreted with caution given the small sample size in the exclusive PD subgroup (*n* = 12). Furthermore, we demonstrated a significant slower overall mean RT (across both image types) in patients with PD compared with HC subjects in the P-MST, but not in the counting Stroop, supporting the notion that SCID observed in the P-MST was due to the child image content rather than due to a general impairment of executive attention. This is further supported by the overall P-MST RT mean difference not retaining significance when adjusting for the PD child SCID.

In contrast to our first main hypothesis, we found a child SCID for participants with PD only, but no adult SCID for HC subjects or patients with PD. In addition, the behavioral results showed no correlation between participants’ sexual orientation and stimuli sex in the P-MST orientation congruence analysis (Section F of the Supplement), which contrasts with previous results from the P-MST (14,37,42), but those studies were not conducted inside an active MRI camera, which could have influenced the results. Prior PD research has shown relatively less sexual (phallometric) differentiation between boys and girls among men with PD, perhaps due to a higher visual homogeneity between prepubertal boys and girls (as compared with women and men) or to a higher influence of age cues in PD, which aligns with the fact that sexual orientation and stimuli sex did not affect RT_Child_ in the PD group (43,44). The lack of adult SCID for the HC subjects may have been because the images did not induce sufficient sexual arousal (as suggested by the P-MST orientation congruence analysis), and/or HC subjects were better at focusing on the task and, therefore, displayed better cognitive control than PD. Although we did not assess subjective arousal or desire ratings, we suggest that the observed behavioral effect in PD may be driven by child versus adult rather than the combinatory effects of child versus adult and sex preferences.

### Implications of the Differential Brain Region Activation

We found an increased activation in the main contrast for PD in regions pertaining to the default mode network (DMN), which broadly encompasses the medial frontal cortex, posterior cingulate, precuneus, angular gyrus, hippocampus, and parts of the parietal cortex; the DMN has been implicated in self-referential thought and thinking of others (emotions of others, social evaluation, and moral reasoning), two factors of putative importance to self-reported and help-seeking patients with PD recruited for this study (45, 46, 47, 48). Structural and functional alterations in this network have previously been implicated in PD (4,49). Disruptions to this network have also been found in autism spectrum disorder (50), which has implications for our study because 62% of patients screened positive for the condition on the RAADS-14 clinical rating scale.

The activation of the medial frontal cortex was confined to its anterior part, primarily the ventral prefrontal and pregenual anterior cingulate regions, and less so dorsomedial prefrontal regions. The outline of the differential activation in PD in our study encompasses regions that are predominantly implicated in decision making, social cognition, and reward processing (51). The anterior medial frontal cortex also coactivates extensively with DMN regions (46), along with limbic regions such as the amygdala and ventral striatum. Differential activation has been implicated in these areas in previous neuroimaging studies of PD (18,19). Consistent with our second main hypothesis, we found differential activation in PD during the main contrast (child > adult) in frontal lobe networks but also in areas related to sexual processing and areas comprising the DMN. In summary, regarding our second hypothesis, we observed increased activation in brain regions involved in functions such as executive attention and decision making but also pertaining to visual perception, emotional processing, self-referential thought, social perception, and sexual arousal. These functional brain abnormalities may explain the behavioral observations. We speculate that in a help-seeking PD sample, child stimuli presented within a task situation centered around PD causes DMN activation (in addition to other areas); this may mediate nonsexual interference in task performance, as suggested (albeit in another context and population) in a previous study (52). This resonates with the suggestion that DMN deactivation facilitates cognitive performance in externally oriented tasks (53). Future studies are needed to establish the mechanisms of the P-MST; one such interesting lead would be to correlate DMN activation to RT. Furthermore, future studies should investigate whether the functional and behavioral patterns outlined in this study hold value as treatment targets, risk markers, and/or for treatment evaluation or prediction in studies with longitudinal designs and correlations to CSA offenses.

### Results in Relation to Previous fMRI Studies in PD

There are two previous cross-sectional case-control studies of PD that have investigated neural activation in the context of executive attention. One study of 10 convicted patients indirectly implicated attention bias and found increased activation of the right amygdala in patients during interference from preferential sexual distractors (28). The authors interpreted the increase as fearful emotion or sexual arousal and suggested a cognitive mechanism by which PD stems from impaired reduction of emotional arousal for children. However, no behavioral data were collected to control for responses, which precludes any direct inference concerning alterations in behavioral control. The second study investigated 9 patients with PD while they performed a concomitant choice reaction time task and found increased activation of the cingulate gyrus and insula together with increased RTs, directly indicating attention bias with alterations in behavioral control (32), which is in agreement with the results of our study.

The differential activation in the child > adult contrast for participants with PD in this study concurs with findings from previous similar studies on PD, implicating the thalamus, insula, hippocampus, caudate, precuneus, and right inferior frontal gyrus (29,30,32). In addition, a previous study reported deactivation in a similar but reversed contrast (boy < man and girl < woman) in the following areas of patients with PD: the caudate, cingulate cortex, insula, thalamus, cerebellum, temporal and occipital cortices, and right inferior frontal gyrus (26). Bilateral activation in the temporal and lateral occipital cortices and the cerebellum coincide with activation by visual child stimuli for men with PD (and adult stimuli for control subjects) in a recent study (21). The caudate, insula, anterior cingulate cortex, cingulate gyrus, temporal and lateral occipital cortices, thalamus, parietal cortex (left postcentral gyrus), and cerebellum, all implicated in our study, have previously been connected to sexual processing and arousal (30,32,54).

### Strengths and Limitations

This study is based on a uniquely recruited help-seeking nonforensic PD sample (6). The sample is large compared with previous PD fMRI studies (6). Notably, to our knowledge, this is the largest single-center study that has investigated neural information processing during self-control using a task paradigm, in patients with PD at risk of committing CSA, with potential ecologic validity (6,32).

The case-control study design does not allow for causal inference. Another limitation is that the counting Stroop and P-MST, while both nominally Stroop tasks, function through different mechanisms, which precludes conclusions based on comparison between them. Future studies should identify how neural activation in PD relates to specific PD subgroups, e.g., those with hyper- or hyposexuality, with different sexual preferences and comorbidities.

### Conclusions

Our results suggest that PD is associated with altered functional brain activity and RT during presentation of sexually salient child images in a test paradigm probing behavioral control. The findings contribute to our understanding of the neurocognitive underpinnings of PD.

## Supplementary Material

Supplementary Material

Key Resources Table
