# Supplementary material for: Altered Neural and Behavioral Response to Sexually Implicit Stimuli During a Pictorial-Modified Stroop Task in Pedophilic Disorder
Source: Biol Psychiatry Glob Open Sci. 2022 Feb 24;3(2):292–300. doi: 10.1016/j.bpsgos.2022.02.004 (PMC10140453; doi:10.1016/j.bpsgos.2022.02.004)
Supplement: Supplementary Material [file mmc1.pdf]

Altered Neural and Behavioral Response to Sexually Implicit Stimuli During a Pictorial-Modified Stroop Task in Pedophilic Disorder

Supplementary Information

Methods and Materials

Section A. Recruitment procedure

The study was performed within the framework of the project Pedophilia at risk: Investigation of Treatment and Biomarkers (PRIOTAB) (1). Each subject provided oral and written informed consent to participate in the study. Patients were help-seeking and self-identified men, 18-66 years old with pedophilic disorder, and were consecutively recruited through a Swedish national helpline (PrevenTell) for people experiencing unwanted or dangerous sexuality. PrevenTell is operated by the research center and multidisciplinary clinic ANOVA, Karolinska University Hospital, Stockholm, Sweden, which specializes in sexual medicine, andrology and transgender medicine. Functional MRI (fMRI), the Pictorial-modified Stroop task (P-MST) and the Counting Stroop data gathering was conducted in Karolinska University Hospital, Huddinge, Sweden whereas the rest of the study was conducted at the ANOVA clinic. Patients were asked to anonymously participate in the study. For further details regarding recruitment see elsewhere (2). Matched healthy controls were recruited from the Stockholm catchment area through Karolinska Trial Alliance and via advertisements on the Karolinska Institute website.

Section B. Participant characterization

Subject characterization included a structured clinical interview for psychiatric diagnosis according to DSM-IV-TR using MINI 6.0 (3), and pedophilic disorder specifically according to DSM-5 criteria (4). Two senior consultants in psychiatry did the clinical

interviews (C.R., and B.L.). We screened for attention-deficit hyperactivity disorder, and substance use disorders with the Adult Attention-Deficit/Hyperactivity Disorder Self-Reporting Rating Scale (ASRS), Alcohol Use Disorders Identification Test (AUDIT) and Drug Use Disorders Identification Test (DUDIT) self-rating scales; respectively (5-7). We evaluated symptoms of autism spectrum disorder using the Ritvo Autism and Aspergers Diagnostic Scale (RAADS-14) using the whole scale score as well as the suggested screening threshold of >13 (8). We assessed levels of sexual desire, and severity of hypo- and hypersexuality with the Sexual Desire Inventory (SDI) and Hypersexual Behavior Inventory (HBI) self-rating scales, respectively (9, 10). We investigated a composite dynamic risk score based on both self- and expert-completed measures of five putative risk domains. For further details about the dynamic risk score and the results for our sample, see elsewhere (1, 2). We determined handedness with the Edinburgh Handedness Scale (11). Neuropsychological functioning was assessed using the Wechsler Adult Intelligence Scale, WAIS-IV (12). We recorded medication status, weight, and height. We present sample characteristics in Table 1, for in-depth analysis and discussion regarding the sample characteristics, see elsewhere (13). One PD (age 36, stated exclusive attraction to female children) and one HC (age 19, heterosexual) participant had missing MRI data, and were therefore excluded from the fMRI analysis. Exclusion reasons can therefore be considered as random. Participant characteristics for the sample included in the fMRI analysis are presented in Table 2. The Counting Stroop sample differed minimally and is described in the Counting Stroop analysis section below (Section E).

Section C. Experimental paradigm

The P-MST and the Counting Stroop were presented using the E-Prime v 2.0 software (Psychology Software Tools, Sharpsburg, PA, USA). Participants were instructed on how to perform the task off-scanner, and performed the task while in the active MRI-scanner,

following instructions and presentation of stimuli on a 32" LCD computer screen (Cambridge Research Systems, Rochester, UK) viewed through a head-coil mounted mirror.

The Pictorial modified Stroop task (P-MST)

In the P-MST, the control trials depicting cats were omitted in the analysis (but were used in a post-hoc analysis) as image content was non-human, and the animals displayed were predatory cats unsuitable for comparison with human stimuli for various reasons, including the possibility of evoking fear (14-16).

The Counting Stroop

The participants completed a Counting Stroop to determine differences in overall mean response time (RT) across both *conditions (Neutral* or *Incongruent)* (17). In the Counting Stroop, participants answer the number of copies (1-4) of a word presented by corresponding button-press, regardless of meaning. During the *Incongruent* condition, the words are numbers that are incongruent with the number of copies presented, such as two copies of the word "three". During the *Neutral* condition, the words are neutral (not expected to evoke sexual or emotional response) objects with a maximum of three syllables. Each trial was presented for 1.5 s in blocks of ten trials each, with alternating *Neutral* and *Incongruent* blocks for a total of sixteen blocks (eight of each condition) in a session. Only one session was administered, with a 30 s fixation spot at the beginning and end. Similarly to the P-MST, participants were instructed to answer both quickly and accurately.

Section D. Brain imaging

After the functional scans had been collected, a T1-weighted anatomical image [magnetization prepared rapid acquisition gradient echo (MP-RAGE)], 176 slices; TR, 1900 ms; TE, 2.52 ms; with an isotropic voxel size of 1 mm × 1 mm × 1 mm] was acquired for all subjects. A senior consultant in neuroradiology assessed the anatomical scans of each subject for pathological signs.

Data processing was carried out using the fMRI Expert Analysis Tool version 6.00. Rigid-body head motion correction was first performed (18). Non-brain tissue was then removed (19) and the functional data was smoothed using a Gaussian kernel set to a full-width half-maximum (FWHM) of 6 mm. To account for time differences in slice acquisition, we performed slice-timing correction using Fourier-space time series phase shifting. In addition, we normalized the grand-mean intensity of the entire four-dimensional (4D) dataset by a single multiplicative factor. We subsequently denoised data using the user-independent and multivariate ICA-based classifier ICA-AROMA (20), set to default non-aggressive denoising. Registration of the data to standard anatomical space was undertaken with high-resolution structural (T1) scans using boundary-based registration with BBR, and non-linear registration and spatial normalization with FNIRT (21, 22). Estimated transformations were subsequently applied to the co-registered functional data. The time series of each subject was modeled using a general linear model (GLM) containing a single predictor representing the on–off time-course of the experiment, convolved with a hemodynamic response function (gamma). In addition, we normalized the grand-mean intensity of the entire four-dimensional (4D) dataset by a single multiplicative factor. Parameter estimates (PEs) were calculated for all brain voxels. Correction for local autocorrelation in the time series was undertaken using FILM (23).

We added age in the regression model to remove its potentially confounding effect. Covariates were mean centered across both groups. For probabilistic anatomical inference we used the standard anatomical atlases supplied with FSL. Lobar structure was reported using the *MNI structural atlas* (24, 25), and single structures with the *Harvard-Oxford cortical and subcortical structural atlas* (26-29) . We report standard-space (MNI152) coordinates of peak activation, *Z*-max, *p*-values, SDs, and number of voxels at the cluster

level ($Z \geq 3.1$, $p = 0.05$). For larger activation clusters that span several anatomical structures of interest we report the six most significant ones.

Section E. Analysis

Demographic and clinical variables

Group differences presented in Table 1 were calculated using IBM SPSS Statistics 25, using *t* tests for normally distributed data, the Mann–Whitney *U* test for skewed data and χ^2-tests for dichotomous variables. Effect size was estimated with Cohen´s *d*, calculated as standardized mean differences for parametric data, via biserial rank correlations for non-parametric data, or from frequency distributions in a 2 by 2 frequency table for categorical data.

fMRI sensitivity analyses controlling for clinical variables and potential confounders

For sensitivity analyses testing for potential confounding factors, we extracted contrast parameter estimates (COPEs) of neural activation averaged over significant clusters obtained in the principal analysis using Featquery in FSL. First, we compared neural activation between groups to validate our main findings. We used the same statistical model as described for fMRI analyses but using the COPE value as a dependent variable in an ANCOVA. We then tested for potential confounding effects by demographic or clinical variables on group differences by entering them as additional covariates (one at a time) in the statistical model. In further sensitivity tests, when fewer than ten participants had a specific comorbidity/medication, we repeated confounder analyses one at a time after excluding those individuals. Specifically, we performed follow-up analyses controlling for body mass index (BMI), handedness, intelligence quotient, sexual abuse victimization before age 15, child sexual offending, adult-related sexual orientation, hyper- and hyposexuality, psychiatric comorbidities, substance use, symptoms of autism spectrum disorder and attention-deficit hyperactivity disorder, treatment with antidepressants and other psychoactive medications.

We performed further analyses after excluding cases with a diagnosis of panic disorder, psychotic syndrome, bipolar disorder, dysthymia, post-traumatic stress disorder, bulimia nervosa, and PD with a pedophilic sexual attraction towards boys only, or both boys and girls, respectively.

Behavioral data preprocessing

RT under 150ms were removed from the analysis to ensure that the recorded RT were not mistakenly carried over from previous trials. 150ms was a conservative arbitrary estimate of minimum required time for processing of the task based on prior data regarding human visual reaction times (30-32), while also keeping in mind that the task requires a processed motor response. Furthermore, RT from when the same color was shown three or more times in a row (such occurrences were unevenly divided between the stimuli categories) was removed from the analysis since pre-analysis showed that they clearly skewed the RT negatively (i.e., led to lower RT). Behavioral data was checked for and fulfilled the requirements for normal distribution. Response data was inspected to make sure the participants performed the task as instructed. One individual from each group was excluded from the P-MST analysis due to this, but it was attributed to their pre-reported color blindness. One HC was excluded from the Counting Stroop due to not performing the task correctly.

Counting Stroop analysis

A 2x2 mixed factorial ANOVA was conducted on the RT variable, with c*ondition* (*Neutral* or *Incongruent*) as the within subjects' factor, and *group* as the between subjects' factor without age as a covariate. However, including age in the model did not change the results. Paired t-test were used to follow up the results and determine within group differences. In this analysis all PD participants were included (same as in Table 1), but only 54 HC were included, due to one HC (age 44) missing input data.

Behavioral secondary analyses (P-MST)

In secondary analyses, we compared *exclusive PD* (*n* = 12, mean age: 40.5 years, SD: 14.3 years) and *non-exclusive PD* (*n* = 38, mean age: 34.1 years, SD: 10.2 years) in a 2x2 ANOVA with the dependent variables *PD-subgroup* (*exclusive* or *non-exclusive)* and *image-type* (adult or child). This subgroup stratification (*exclusive PD* or *non-exclusive PD*) was based on whether a patient's stated sexual attraction was exclusive to prepubescent children, or to both adults and children. We use the same follow-up approach (paired t-tests) as in the main analysis.

To determine how preferential sexual stimuli affect the SCID, we also tested the effects of sexual orientation and its relation to stimuli sex in both groups, using a 2x2x2 ANOVA. We refer to this analysis as the *P-MST orientation congruence analysis*, with only homo- and heterosexual individuals included as it required a single coherent sex preference for both child and adults. Dependent variables were *group* (HC or PD), *image-type* (adult or child), and sexual orientation congruence with stimuli sex, *sexual orientation congruence,* defined as either sexual orientation congruent with stimuli sex (*SexualOrientation+*) or sexual orientation incongruent with stimuli sex (*SexualOrientation-*). We coded for stimuli sexual orientation congruence (+) or incongruence (-) according to self-reported hetero- or homosexuality in relation to the sex of the person presented in the images (*female*, consisting of girls and women or *male*, consisting of boys and men), while excluding those with both female and male preferences from the analysis. For example, for a heterosexual (male) participant, stimuli presenting females were categorized as sexual orientation congruent (*SexualOrientation+*), whereas stimuli presenting males as sexual orientation incongruent (*SexualOrientation-*). Crossing sexual orientation congruence with image-type (adult or child) results in four variable categories: *AdultSexualOrientation+*, *AdultSexualOrientation-*, *ChildSexualOrientation+*, and *ChildSexualOrientation-*). For example, for a heterosexual

(male) participant, pictures depicting women were categorized as *AdultSexualOrientation+*, while pictures of boys were classified as *ChildSexualOrientation-*. The RT from these four variables were then entered into the 2x2x2 ANOVA, together with the two-level variable *group*. In this analysis, 41 PD participants (mean age: 36.5 years, SD: 12.2 years) were included, of whom 35 self-identified as heterosexual and six as homosexual, in addition to 49 HC participants (mean age: 35.9 years, SD: 11.6 years), of whom 45 self-identified as heterosexual and four as homosexual.

To investigate the significant group interaction in the main behavioural analysis as a relative percentual change, a Univariate analysis was conducted on a measure obtained by dividing the RT adult-child difference by RT adult (*pct-RT* = $\Delta RT/RT_{Adult}$).

Furthermore, to determine whether child stimuli was driving the overall mean (across both image-types) RT group difference in the P-MST, a post-hoc analysis was conducted. In the post-hoc analysis we used independent sample t-tests to compare the RT between groups in the *control* (cats) stimuli category as well as two composite measures, one with all the RT (Cats + Adults + Children, *CACh*) and one other where we removed the child input (Cats + Adults, *CA*). This was done to account for the influence of the increased PD child RT (SCID) on the overall mean RT difference.

Results

Section F. Behavioral results

Counting Stroop

The 2x2 mixed factorial ANOVA yielded a significant main effect of *condition* (Neutral M = 740.59 ± 75.06 ms; Incongruent M = 757.89 ± 76.65 ms; F (1, 103) = 23.084 $p < 0.001$ $Eta^2_p = 0.183$). There was a non-significant main effect of *group* (HC M = 745.88 ± 73.55 ms; PD M = 752.59 ± 73.55 ms; F (1, 103) = 0.218 $p = 0.641$ $Eta^2_p = 0.002$). The *condition* × *group* interaction was non-significant (F (1, 103) = 2.012 $p = 0.159$ $Eta^2_p =$

0.019). For reasons of completeness, we present the results of follow-up paired t-tests. Paired t-tests within the groups revealed a significant difference in the PD group between the Neutral (M = 746.49 ms, SD = 80.15 ms) and Incongruent conditions (M = 758.69 ms, SD = 83.02 ms; *Cohen´s d* = 0.340 $p = 0.019$ t (50) = -2.430). Within the HC group, there was also a significant difference between the Neutral (M = 734.68 ms, SD = 69.84 ms) and Incongruent conditions (M = 757.09 ms, SD = 70.04 ms; *Cohen´s d* = 0.593 $p < 0.001$ t (53) = -4.351). These results are illustrated by Figure S1.

P-MST, exclusive vs non-exclusive PD

We found statistically significant effects of *image-type* (RT_{Adult} = 829.83 ± 104.36 ms; RT_{Child} = 859.71 ± 106.04 ms; F (1, 48) = 11.274, $p = 0.002$ $Eta^2_p = 0.190$) and no significant effects of *PD-subgroup* ($RT_{Exclusive}$ = 859.34 ± 85.75 ms; $RT_{Non\text{-}exclusive}$ = 830.20 ± 85.75 ms; F (1, 48) = 1.053, $p = 0.310$ $Eta^2_p = 0.021$), or the *image-type* × *PD-subgroup* interaction (F (1, 48) = 2.581, $p = 0.115$ $Eta^2_p = 0.051$). Although the interaction was not significant, we present results of paired t-tests for completeness. Paired t-tests revealed significant differences in RT between the *child* and *adult image-types* in the *exclusive PD* group (M $RT_{Adult\_exclusive}$ = 837.24 ms, SD = 78.15 ms; M $RT_{Child\_exclusive}$ = 881.42 ms, SD = 71.73 ms; Cohen's $d = 1.378$, $p = 0.001$, t (11) = -4.775) and a non-significant difference within the *non-exclusive PD* group (M $RT_{Adult\_non\text{-}exclusive}$ = 822.41 ms, SD = 92.16 ms; M $RT_{Child\_non\text{-}exclusive}$ = 837.99 ms, SD = 95.46 ms; Cohen´s $d = 0.266$, $p = 0.110$, t (37) = -1.637). These results are illustrated in Figure S2.

P-MST orientation congruence analysis

In the 2×2×2 ANOVA, we found no significant effects. *Orientation congruence* (RT_{Pref} = 820.07 ± 82.87 ms; $RT_{Non\text{-}Pref}$ = 818.75 ± 87.33 ms; F (1,88) = 0.057, $p = 0.812$ $Eta^2_p = 0.001$), the *orientation congruence* × *group* interaction (F (1, 88) = 0.088, $p = 0.768$ $Eta^2_p = 0.001$), the *image-type* × *orientation congruence* interaction (F (1, 88) = 0.684, p =

0.410 $Eta^2_p = 0.008$) and the *image-type × orientation congruence × group* interaction (F (1, 88) = 0.059, $p = 0.829$ $Eta^2_p = 0.001$) were all non-significant. These results are illustrated by Figure S3.

Behavioral results (P-MST, pct-RT = $\Delta RT/RT_{Adult}$)

The Univariate analysis using the pct-RT variable yielded a significant difference between the PD (M = -0.029 ± 0.0686) and HC (M = -0.0025 ± 0.0468; F (1, 102) = 5.492 p = 0.021)

Post-hoc behavioral results (testing the P-MST overall mean RT difference)

Independent sample t-tests yielded a non-significant difference in the *control* stimuli category (M RT_{Cats_PD} = 778.89ms, SD = 77.68 ms; M RT_{Cats_HC} = 755.11 ms, SD = 75.45; Cohen´s d = 0.311 p = 0.116 t (102) = 1.583), the *CACh* measure (M RT_{CACh_PD} = 807.26 ms, SD = 79.10 ms; M RT_{CACh_HC} = 777.47 ms, SD = 75.28 ms; Cohen´s d = 0.386, p = 0.052, t (102) = 1.968) and the *CA* measure (M RT_{CA_PD} = 794.54 ms, SD = 79.07 ms; M RT_{CA_HC} = 770.04 ms, SD = 76.02 ms; Cohen´s d = 0.316, p = 0.110, t (102) = 1.611.

Section G. Results of the fMRI sensitivity analysis

fMRI sensitivity analyses controlling for clinical variables and potential confounders

For the fMRI-derived measures, results for cluster 1-9 remained robust when correcting for all demographic and clinical variables.

Figure S1. Mean response time (RT) in the Counting Stroop for pedophilic disorder (PD) and healthy control (HC) participants, for the Neutral and Incongruent conditions.

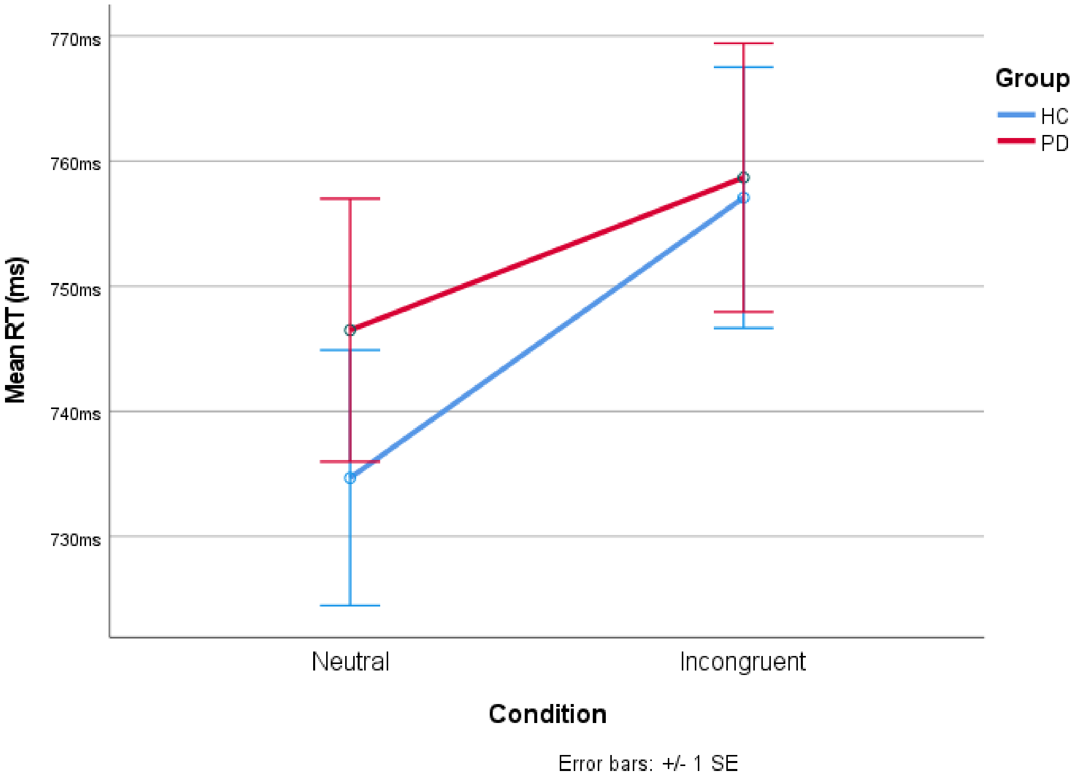

Figure S2. Mean response time (RT) in the Pictorial-modified Stoop task for the *exclusive* pedophilic disorder (*PD)* and *non-exclusive PD* groups for the a*dult* and *child* image-types. * indicates a statistically significant difference (*$p < 0.05$*).

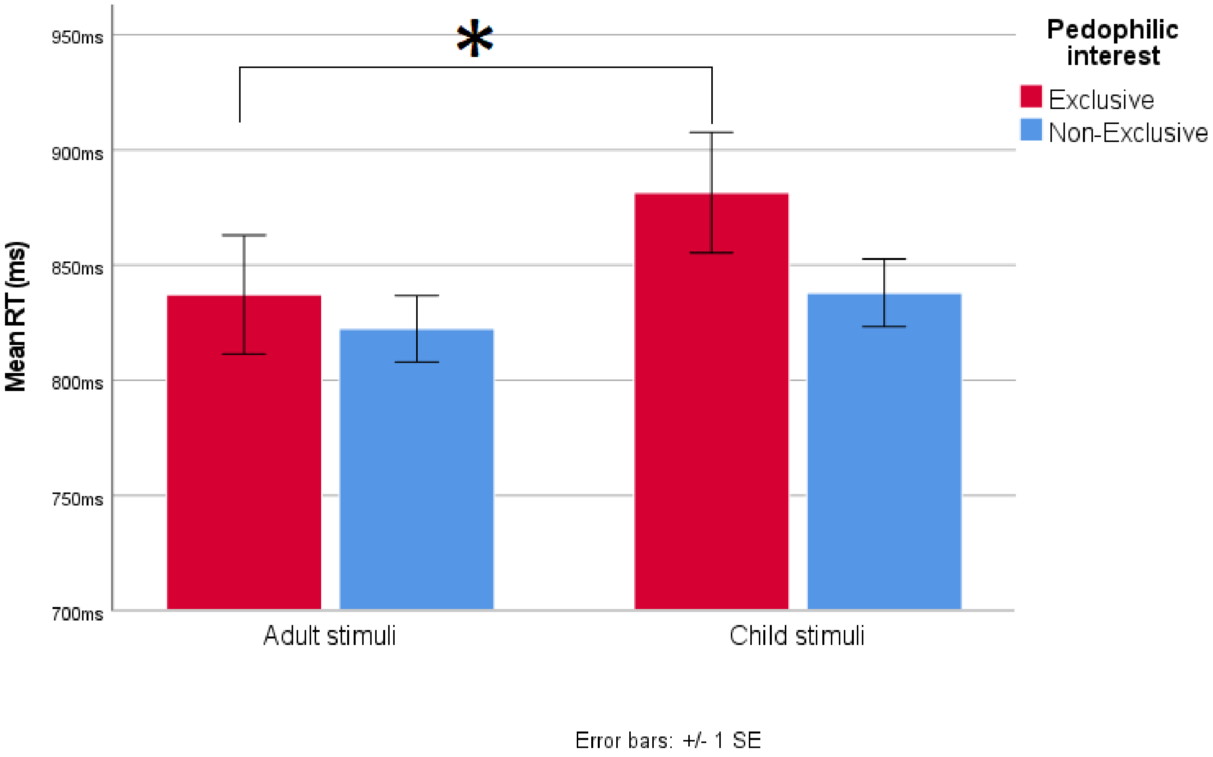

Figure S3. Mean response time (RT) for healthy controls (HC) and pedophilic disorder (PD) patients when responding to the four different *adult* and *child* variables, adjusted according to stimuli sex and participant´s sexual orientation. This is based on if stimuli´s sex is congruent with the sex preference of the subject´s sexual orientation. *SexualOrientation+ = Sexual orientation congruent. SexualOrientation- = Sexual orientation incongruent*

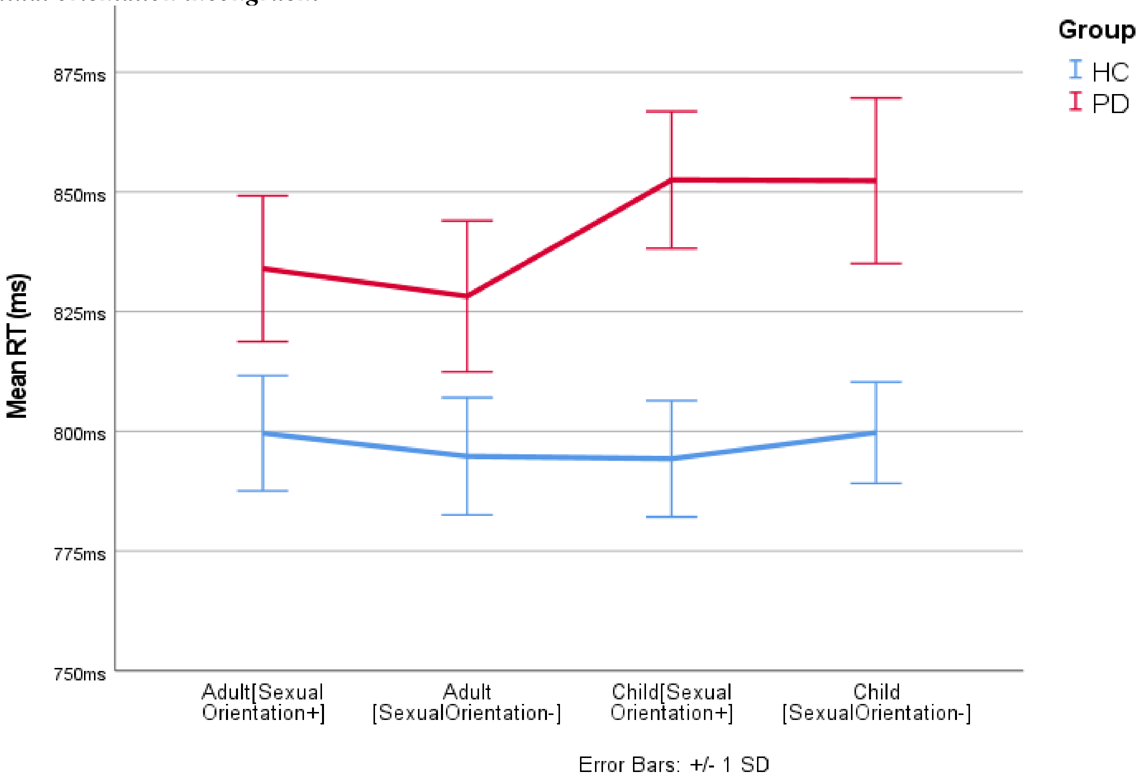

Figure S4. Mean group activation pattern in pedophilic disorder patients for the *child>adult* contrast.

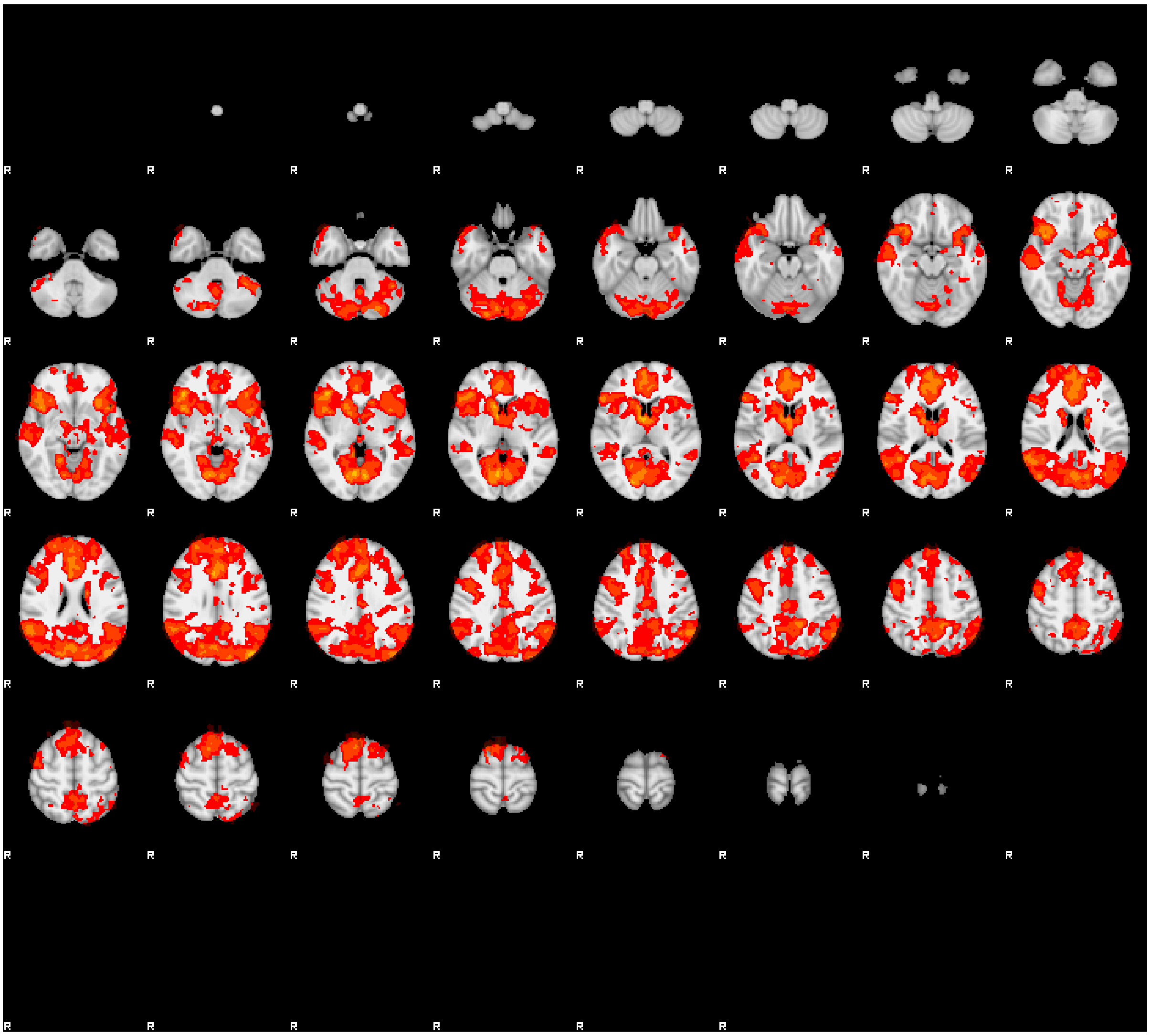

Figure S5. Mean group activation pattern in healthy controls for the *child>adult* contrast.

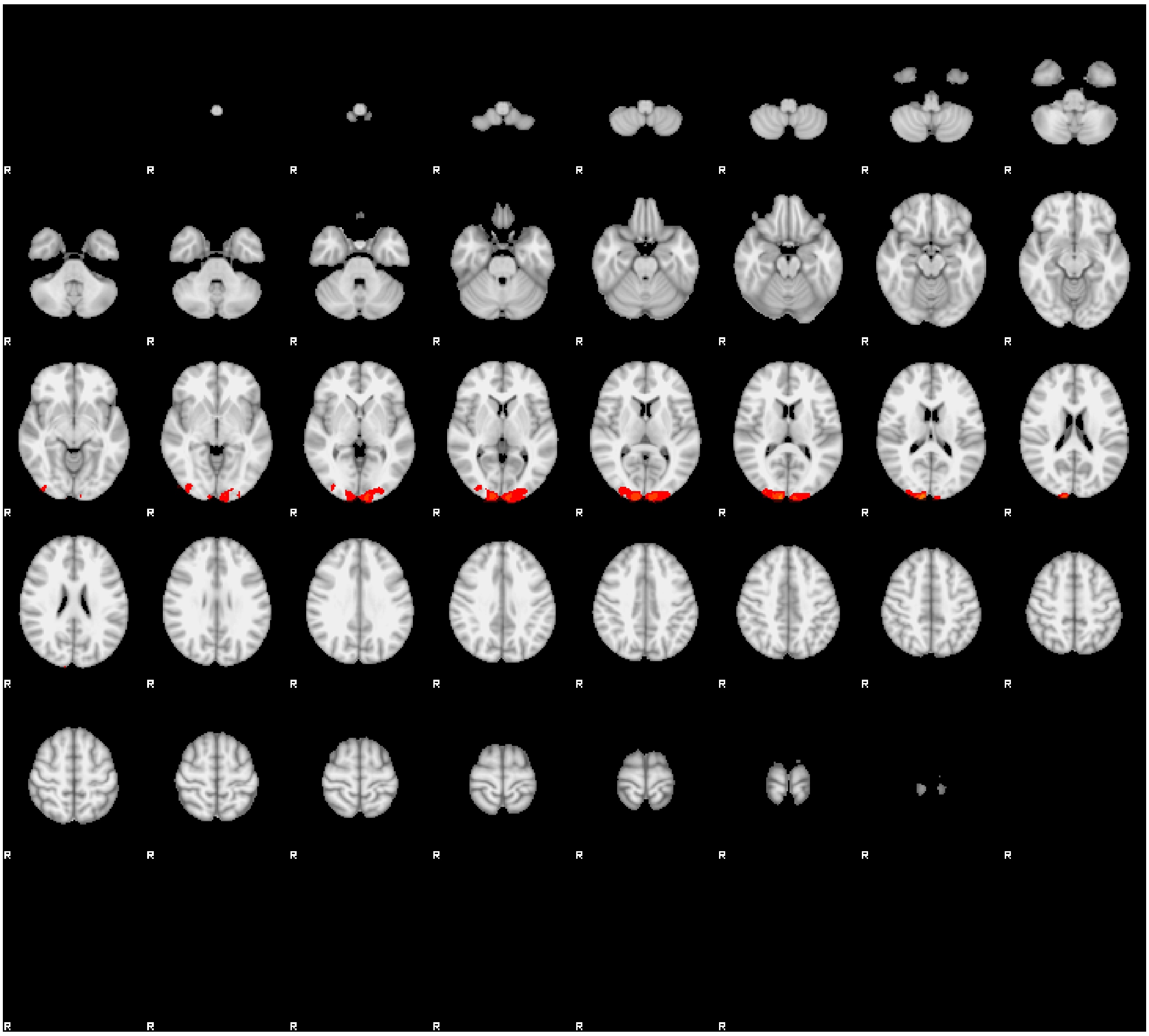

Table S1. List of 9 clusters with increased functional activation in pedophilic disorder compared to healthy controls. For local maxima within those clusters, see Table S2.

Cluster #	Voxels	-log$_{10}$(*P*)	*Z*	*Z*-max (MNI)			COPE	MNI Atlas	Harvard-Oxford Cortical Structural Atlas
				X	Y	Z			
9	20051	69.7	6.04	-14	-86	26	21.9	55 % Occipital Lobe	18 % Lateral Occipital Cortex, superior division; 8 % Cuneal Cortex
8	5187	27.6	4.99	-8	44	14	23.2	76 % Frontal Lobe	51 % Paracingulate Gyrus; 29 % Cingulate Gyrus
7	1179	9.48	4.77	42	-50	12	13.5	5 % Temporal Lobe	9 % Angular Gyrus; 9 % Middle Temporal Gyrus
6	1098	8.99	5.8	40	14	-12	15.9	50 % Insula	69 % Insular Cortex; 4 % Frontal Orbital Cortex
5	385	3.84	4.58	-30	16	-14	21.9	4 % Insula	40 % Frontal Orbital Cortex; 40 % Insular Cortex
4	328	3.33	4.27	36	-6	36	10.5	-	1 % Precentral Gyrus
3	313	3.19	4.13	-56	-6	-6	13.5	74 % Temporal Lobe	56 % Superior Temporal Gyrus; 5 % Middle Temporal Gyrus
2	235	2.42	4.96	-32	-26	32	8.62	-	-
1	185	1.88	4.92	42	32	-2	12	42 % Frontal Lobe	24 % Frontal Orbital Cortex; 9 % Inferior Frontal Gyrus

Table S2. List of increased functional activation in pedophilic disorder compared to healthy controls (local maxima, n = 6) for the 9 significant clusters

Cluster #	*Z*	x	y	z	Harvard-Oxford Atlas
9	6.04	-14	-86	26	18 % Lateral Occipital Cortex, superior division
	5.53	42	-72	-4	53 % Lateral Occipital Cortex, inferior division
	5.43	20	-82	-20	20 % Occipital Fusiform Gyrus
	5.38	-34	-44	-16	24 % Temporal Occipital Fusiform Cortex, posterior division
	5.17	-18	-78	44	61 % Lateral Occipital Cortex
	5.11	-22	-30	-8	60 % Hippocampus Subiculum L
8	4.99	-8	44	14	51 % Paracingulate Gyrus
	4.98	-2	34	6	65 % Cingulate Gyrus, anterior division
	4.98	6	40	-12	43 % Paracingulate Gyrus
	4.96	4	-4	8	86 % Thalamus
	4.85	-14	38	-6	5 % Paracingulate Gyrus
	4.85	10	40	4	39 % Cingulate Gyrus, anterior division
7	4.77	42	-50	12	23 % Angular Gyrus
	4.62	36	-38	8	White Matter
	4.45	48	-52	32	32 % Angular Gyrus
	4.39	40	-48	18	10 % Angular Gyrus
	4.37	54	-56	24	71 % Angular Gyrus
	4.3	54	-58	20	46 % Angular Gyrus
6	5.8	40	14	-12	69 % Insular Cortex
	4.86	44	28	-32	6 % Temporal Pole

	4.62	66	-2	-18	41 % Middle Temporal Gyrus, anterior division
	4.51	62	-10	-14	40 % Middle Temporal Gyrus, posterior division
	4.34	52	22	-18	30 % Temporal Pole
	4.27	64	-4	-10	30 % Middle Temporal Gyrus, anterior division
5	4.58	-30	16	-14	40 % Frontal Orbital Cortex
	4.27	-32	6	-18	19 % Temporal Pole
	4.23	-40	10	-8	94 % Insular Cortex
	3.57	-28	16	-2	3 % Insular Cortex
	4.58	-30	16	-14	40 % Frontal Orbital Cortex, 40 % Insular Cortex
	4.27	-32	6	-18	19 % Temporal Pole, 12 % Insular Cortex
4	4.27	36	-6	36	1 % Precentral Gyrus
	4	40	4	22	7 % Precentral Gyrus
	3.78	42	-4	34	12 % Precentral gyrus
	3.54	40	14	24	39 % Inferior Frontal Gyrus, pars opercularis
	3.5	40	2	44	40 % Middle Frontal Gyrus
	3.33	42	6	30	34 % Precentral Gyrus
3	4.13	-56	-6	-6	56 % Superior Temporal Gyrus, anterior division
	4.11	-62	-4	-18	66 % Middle Temporal Gyrus, anterior division
	3.83	-64	-20	-10	68 % Middle Temporal Gyrus, posterior division
	3.76	-64	0	-22	17 % Middle Temporal Gyrus, anterior division
	3.71	-58	-12	-14	35 % Middle Temporal Gyrus, posterior division

	3.4	-62	6	-14	5 % Temporal Pole
2	4.96	-32	-26	32	White Matter
	3.84	-22	-30	34	WM
	3.57	-26	-32	28	WM
1	4.92	42	32	-2	24 % Frontal Orbital Cortex
	3.67	32	34	-2	7 % Frontal Orbital Cortex
	3.53	24	44	-6	5 % Frontal Pole
	3.46	28	36	-6	16 % Frontal Orbital Cortex

References

1. Landgren V, Malki K, Bottai M, Arver S, Rahm C (2020): Effect of Gonadotropin-Releasing Hormone Antagonist on Risk of Committing Child Sexual Abuse in Men With Pedophilic Disorder: A Randomized Clinical Trial. *JAMA Psychiatry*. 77:897-905.
2. Wittström F, Långström N, Landgren V, Rahm C (2020): Risk Factors for Sexual Offending in Self-Referred Men With Pedophilic Disorder: A Swedish Case-Control Study. *Front Psychol*. 11:571775.
3. Sheehan DV, Lecrubier Y, Sheehan KH, Amorim P, Janavs J, Weiller E, et al. (1998): The Mini-International Neuropsychiatric Interview (MINI): the development and validation of a structured diagnostic psychiatric interview for DSM-IV and ICD-10. *Journal of clinical psychiatry*. 59:22-33.
4. American Psychiatric A (2013): *Diagnostic and statistical manual of mental disorders (DSM-5®)*. American Psychiatric Pub.
5. Kessler RC, Adler L, Ames M, Demler O, Faraone S, Hiripi EVA, et al. (2005): The World Health Organization Adult ADHD Self-Report Scale (ASRS): a short screening scale for use in the general population. *Psychological medicine*. 35:245.
6. Bohn MJ, Babor TF, Kranzler HR (1995): The Alcohol Use Disorders Identification Test (AUDIT): validation of a screening instrument for use in medical settings. *Journal of studies on alcohol*. 56:423-432.
7. Berman AH, Bergman H, Palmstierna T, Schlyter F (2005): Evaluation of the Drug Use Disorders Identification Test (DUDIT) in criminal justice and detoxification settings and in a Swedish population sample. *European addiction research*. 11:22-31.
8. Eriksson JM, Andersen LMJ, Bejerot S (2013): RAADS-14 Screen: validity of a screening tool for autism spectrum disorder in an adult psychiatric population. *Molecular Autism*. 4:1-11.
9. Reid RC, Garos S, Carpenter BN (2011): Reliability, validity, and psychometric development of the Hypersexual Behavior Inventory in an outpatient sample of men. *Sexual Addiction & Compulsivity*. 18:30-51.
10. Spector IP, Carey MP, Steinberg L (1996): The Sexual Desire Inventory: Development, factor structure, and evidence of reliability. *Journal of sex & marital therapy*. 22:175-190.
11. Oldfield RC (1971): The assessment and analysis of handedness: the Edinburgh inventory. *Neuropsychologia*. 9:97-113.
12. Weiss LG, Saklofske DH, Coalson D, Raiford SE (2010): *WAIS-IV clinical use and interpretation: Scientist-practitioner perspectives*. Academic Press.
13. Abé C, Adebahr R, Liberg B, Mannfolk C, Lebedev A, Eriksson J, et al. (2021): Brain structure and clinical profile point to neurodevelopmental factors involved in pedophilic disorder. *Acta Psychiatrica Scandinavica*. 143:363-374.
14. Watts FN, McKenna FP, Sharrock R, Trezise L (1986): Colour naming of phobia-related words. *Br J Psychol*. 77 (Pt 1):97-108.
15. Constantine R, McNally RJ, Hornig CD (2001): Snake Fear and the Pictorial Emotional Stroop Paradigm. *Cognitive Therapy and Research*. 25:757-764.
16. Pravossoudovitch K, Cury F, Young SG, Elliot AJ (2014): Is red the colour of danger? Testing an implicit red–danger association. *Ergonomics*. 57:503-510.
17. Bush G, Whalen PJ, Rosen BR, Jenike MA, McInerney SC, Rauch SL (1998): The counting Stroop: an interference task specialized for functional neuroimaging--validation study with functional MRI. *Hum Brain Mapp*. 6:270-282.
18. Jenkinson M, Bannister P, Brady M, Smith S (2002): Improved optimization for the robust and accurate linear registration and motion correction of brain images. *Neuroimage*. 17:825-841.
19. Smith SM (2002): Fast robust automated brain extraction. *Human brain mapping*. 17:143-155.
20. Pruim RHR, Mennes M, van Rooij D, Llera A, Buitelaar JK, Beckmann CF (2015): ICA-AROMA: A robust ICA-based strategy for removing motion artifacts from fMRI data. *Neuroimage*. 112:267-277.
21. Jenkinson M, Beckmann CF, Behrens TEJ, Woolrich MW, Smith SM (2012): Fsl. *Neuroimage*. 62:782-790.

22. Andersson JLR, Jenkinson M, Smith S (2007): Non-linear registration, aka Spatial normalisation FMRIB technical report TR07JA2. *FMRIB Analysis Group of the University of Oxford*. 2:e21.
23. Smith SM, Jenkinson M, Woolrich MW, Beckmann CF, Behrens TEJ, Johansen-Berg H, et al. (2004): Advances in functional and structural MR image analysis and implementation as FSL. *NeuroImage*. 23:S208-S219.
24. Mazziotta J, Toga A, Evans A, Fox P, Lancaster J, Zilles K, et al. (2001): A probabilistic atlas and reference system for the human brain: International Consortium for Brain Mapping (ICBM). *Philosophical Transactions of the Royal Society of London Series B: Biological Sciences*. 356:1293-1322.
25. Collins DL, Holmes CJ, Peters TM, Evans AC (1995): Automatic 3-D model-based neuroanatomical segmentation. *Human brain mapping*. 3:190-208.
26. Makris N, Goldstein JM, Kennedy D, Hodge SM, Caviness VS, Faraone SV, et al. (2006): Decreased volume of left and total anterior insular lobule in schizophrenia. *Schizophrenia research*. 83:155-171.
27. Frazier JA, Chiu S, Breeze JL, Makris N, Lange N, Kennedy DN, et al. (2005): Structural brain magnetic resonance imaging of limbic and thalamic volumes in pediatric bipolar disorder. *American Journal of Psychiatry*. 162:1256-1265.
28. Desikan RS, Ségonne F, Fischl B, Quinn BT, Dickerson BC, Blacker D, et al. (2006): An automated labeling system for subdividing the human cerebral cortex on MRI scans into gyral based regions of interest. *Neuroimage*. 31:968-980.
29. Goldstein JM, Seidman LJ, Makris N, Ahern T, O'Brien LM, Caviness Jr VS, et al. (2007): Hypothalamic abnormalities in schizophrenia: sex effects and genetic vulnerability. *Biological psychiatry*. 61:935-945.
30. Kosinski RJ (2008): A literature review on reaction time. *Clemson University*. 10.
31. Badwe N, Patil K, Yelam S, Vikhe B, Vatve M (2012): A comparative study of hand reaction time to visual stimuli in students of 1st MBBS of a Rural Medical College. *Pravara Medical Review*. 4.
32. Jain A, Bansal R, Kumar A, Singh KD (2015): A comparative study of visual and auditory reaction times on the basis of gender and physical activity levels of medical first year students. *Int J Appl Basic Med Res*. 5:124-127.
